# Novel Concept of Gas Sensitivity Characterization of Materials Suited for Implementation in FET-Based Gas Sensors

**DOI:** 10.1186/s11671-016-1687-z

**Published:** 2016-11-02

**Authors:** Yosri Ayadi, Lama Rahhal, Bertrand Vilquin, Céline Chevalier, Fabian Ambriz Vargas, Serge Ecoffey, Andreas Ruediger, Andranik Sarkissian, Stéphane Monfray, Jean-Pierre Cloarec, Dominique Drouin, Abdelkader Souifi

**Affiliations:** 1 Laboratoire Nanotechnologies Nanosystemes (LN2) - CNRS UMI-3463, Universite de Sherbrooke, 3000 Boul. Universite, Sherbrooke, J1K OA5 Quebec Canada; 2 Institut Interdisciplinaire d’Innovation Technologique (3IT), Universite de Sherbrooke, 3000 Boul. Universite, Sherbrooke, J1K OA5 Quebec Canada; 3 Institut des Nanotechnologies de Lyon (INL) - UMR CNRS 5270, 7 av. Jean Capelle, 69621 Villeurbanne cedex, France; 4 Institut National de la Recherche Scientifique, 1650, Blvd. Lionel-Boulet, Varennes, J3X 1S2 Quebec Canada; 5 Plasmionique Inc, 171B-1650, Blvd. Lionel-Boulet, Varennes, J3X 1S2 Quebec Canada; 6 ST Microelectronics, 850 Rue Jean Monnet, 38920 Crolles, France

**Keywords:** Gas sensor, Field-effect transistor, Surface charge measurement, Hydrogen detection, Platinum

## Abstract

We propose a novel technique to investigate the gas sensitivity of materials for implementation in field-effect transistor-based gas sensors. Our technique is based on the measurement of the surface charge induced by gas species adsorption, using an electrometer. Platinum sensitivity to hydrogen diluted in synthetic air has been evaluated with the proposed charge measurement technique in the operation temperature range from 80 to 190 °C at constant H_2_ concentration of 4 % and for different concentrations ranging from 0.5 to 4 % at 130 °C.

## Background

The two mechanisms associated with gas molecule adsorption on solids are chemisorption run by ionic bonds and physisorption run by Van der Waals interactions. These interactions induce a modification of surface charge and hence surface potential. The effect is stronger in case of chemisorption compared to physisorption, since a partial charge transfer occurs and adsorbates are in a charged state. This leads to a change in material work function [1, 2]. If the inner surface of field-effect transistor (FET) gate is accessible to gas species, the induced voltage shift associated to the work function variation acts as an artificial voltage which is added to an externally applied gate voltage and affects the conductance of the transistor channel [1, 2].

This has been achieved by either a permeable gate electrode made of catalytic metals, in so-called Lundstrom MOSFETs (catalytic MOSFETs), or a suspended gate with a sensitive layer deposited on the outer surface, in suspended gate-FET (SG-FET)-based gas sensors [1, 2]. Since adsorbed species are in a dynamic equilibrium with the gas phase [3, 4], and since the voltage shift is proportional to the number of adsorbed gas species per surface unit, work function variation measurements can be used to monitor the gas concentration.

Catalytic MOSFETs are based on a conventional MOSFET where a catalytic metal, typically Pd, Pt, or Ir, is used usually as a very thin layer (tens of nanometers) to form the gate electrode [4–7]. They, usually operate at temperatures in the range of 100 to 200 °C [6]. They are limited to detection of H_2_ and hydrogen containing gases, such as ammonia, hydrogen sulfide, ethylene, and ethanol [5, 8], since these molecules can be adsorbed and dissociated on the surface of catalytic metals. Released hydrogen atoms diffuse through the catalytic metal layer, which acts as a hydrogen filter, to reach the inner metal surface-oxide interface.

In contrast, SG-FET-based gas sensors have been demonstrated to detect a broad range of gases [1, 2] such as CO [9, 10] and H_2_ [1, 11], thanks to real flexibility in sensitive layer choice and integration, and operate at room temperature or slightly above.

Integration of these sensors is complex mainly because of the necessity to implement a suspended gate electrode with the sensitive material deposited on the back side. The transistor channel width to length ratio has to be increased to overcome the poor transconductance due to the very low capacitance across the air gap [1, 12], which is a limitation towards high integration density. An alternative to these limitations is to use a double gate-FET (DG-FET) as a transducer. The general concept is to incorporate a functionalized gate with a dedicated gas-sensitive material and a control gate. The functionalized gate is a floating gate while the control gate is biased to control the operation point of the transistor. Such device has a greater transconductance. The subthreshold slope of such transistor is of great interest because it allows a greater current variation for functionalized gate potential variation when biased to operate in the linear region of the transfer characteristics.

**Fig. 1:**
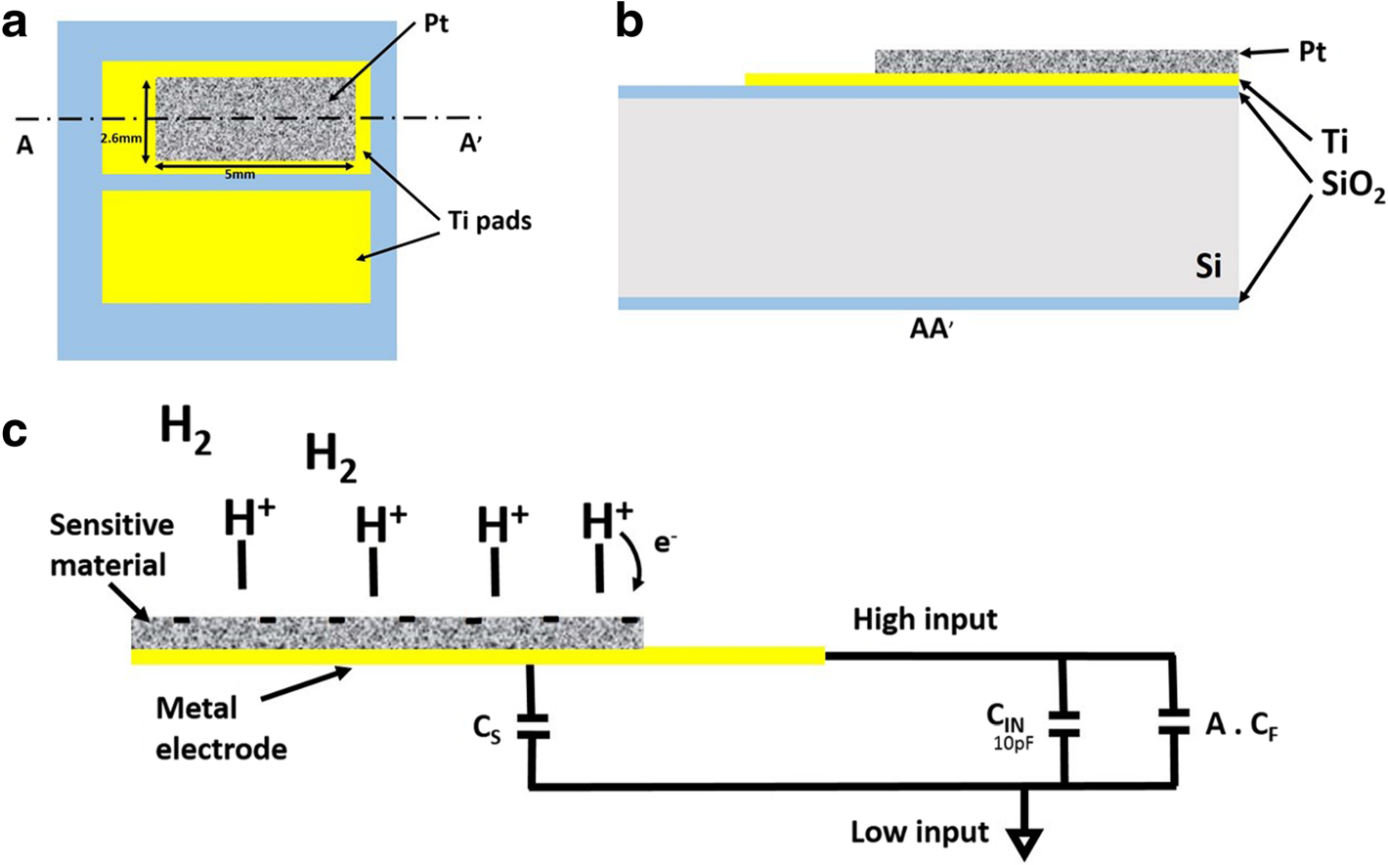
**a** Schematic representation of surface charge measurement configuration and equivalent circuit of sample connected the electrometer with feedback amplifier in Coulomb mode, with the three capacitors *C*_s_, *C*_IN_, and *A C*_f_ in parallel. Chemisorption of H_2_ on platinum is illustrated. **b** Top-view and **c** cross-section schematic representation of sample structure

When hydrogen molecules reach a Pt surface, they dissociate and adsorb on the form of single atoms [2]. Chemisorption of H_2_ on polycrystalline Pt is accompanied with a partial electron transfer into it, and adsorbed atomic hydrogens form a dipole surface layer [2], as shown in Fig. 1a, causing a potential drop at the surface. Platinum has been used to detect hydrogen either with catalytic MOSFETs [5, 6, 13] or in combination with SG-FETs [1, 14].

In this work, we demonstrate a new technique to investigate gas sensitivity of materials based on measuring surface charge variations induced by gas molecule adsorption. Surface charge measurement technique has been applied to investigate chemisorption of hydrogen on platinum surface in synthetic air. We have shown that using our proposed technique, sensitivity can be estimated for optimal sizing of sensing layer surface.

## Methods

### Surface Charge Measurement

Kelvin probe (KP) has been the privileged technique for the investigation of FET-based gas sensor sensitive material. Material sensitivity is given in terms of work function variation induced by gas species adsorption. The output response of conventional SG-FET-based gas sensors is the shift in the channel conductance, usually recorded as a shift in the transistor threshold voltage. This shift is the work function variation induced by the target gas molecule adsorption, divided by the elementary charge. Our characterization technique is based on measuring the material surface charge/potential variation induced by gas molecules adsorption. The technique gives the material surface charge measured in gas environment. Knowing the surface charge variation of the floating functionalized gate of a DG-FET, the channel conductance variation can be estimated.

The proposed technique is easy to implement. Only the sample has to be placed inside a controlled atmosphere cell. In contrast to kP technique, the gas chamber has to be large enough to hold the entire probe setup. A small cell makes it easy to control gas diffusion homogeneity and turbulences in comparison to larger gas chamber. In addition, in KP technique, the work function of the reference probe may be sensitive to the target gas and be a source of error by contributing to the obtained signal.

Charge measurement is carried out using an electrometer with feedback amplifier configuration (Keithley model 6514 electrometer), operated in Coulomb mode. The sensitive layer is deposited on top of a metal electrode that is connected to electrometer high input. The low input is connected to the substrate backside. An electrometer in Coulomb mode is modeled equivalent to two capacitors in parallel: (i) an input capacitance (*C*_IN_) of 10 pF and (ii) an amplifier input capacitance of *A C*_f_. *C*_f_ is the electrometer reference feedback capacitance (capacitor which provides the feedback path around the operational amplifier) of 1000 pF, and *A* is the open loop gain of the operation amplifier. The surface charge measurement configuration and equivalent circuit of the electrometer in Coulomb mode is depicted in Fig. 1a. Since *A* is large enough (*A* = 55 × 10^6^), the product *A C*_f_ dominates all other capacitances *C*_s_ and *C*_IN_. Thus, charge to be measured is completely transferred to the coulombmeter input capacitor *A C*_f_. The electrometer with feedback amplifier configuration allows charge measurements independent of the source capacitance.

### Sample Fabrication

Figure 1b, c illustrates, respectively, a top-view and a cross-section structure of the final device. A 150-nm oxide is thermally grown over a Si substrate. A 50-nm Ti film is deposited by e-beam evaporation and patterned using a UV lift-off process. A Pt sensitive film is then patterned by UV lift-off and deposited by e-beam evaporation on one of the two Ti electrodes. The measured Pt surface area is 13 mm^2^. The Ti electrode beneath the Pt layer is wirebonded to a chip carrier using Al wires. The second Ti electrode serves as a reference to measure its sensitivity to the target gas.

### Sensitive Material Characterization

**Fig. 2:**
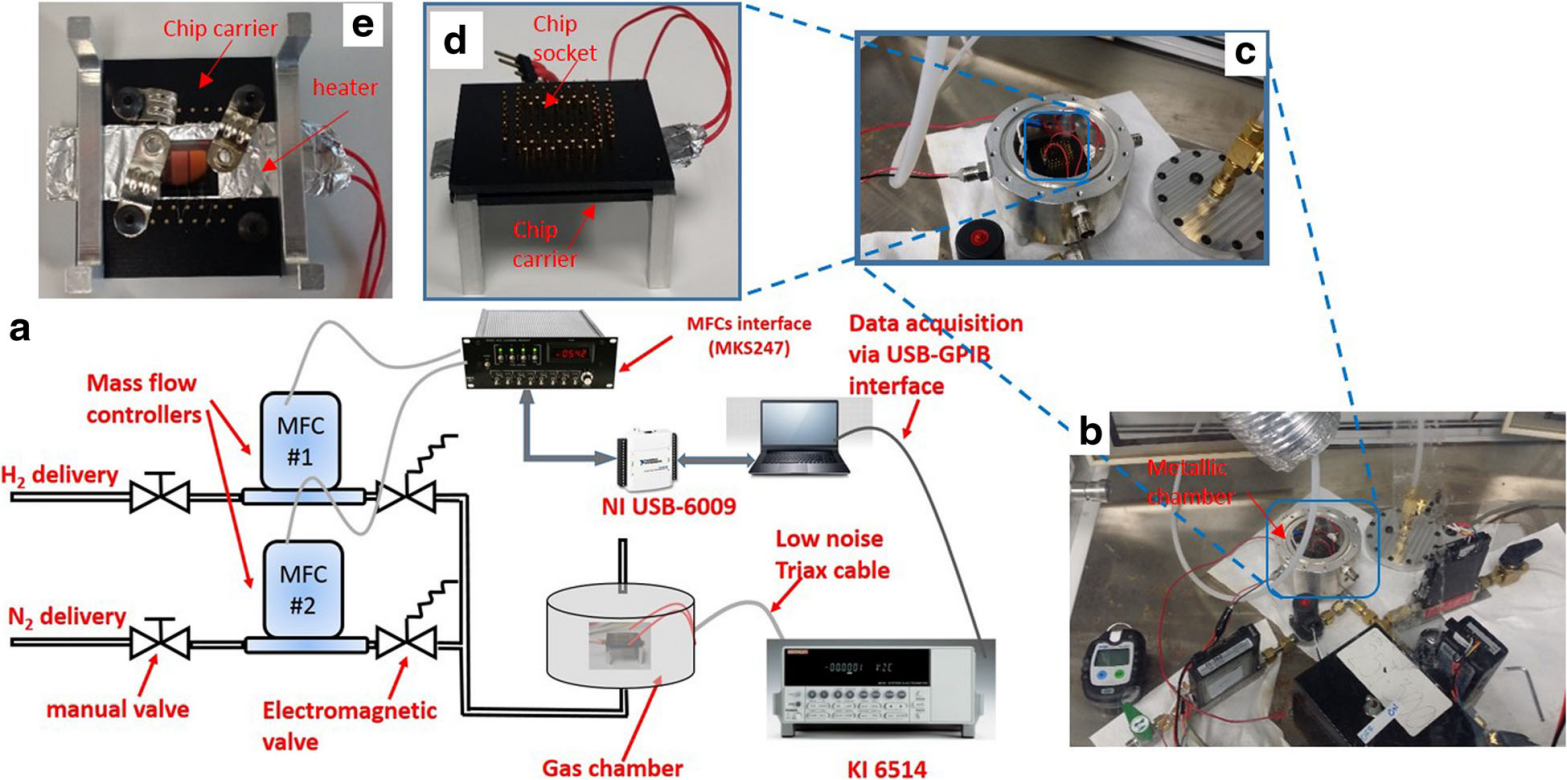
**a** Schematic of the experimental setup. **b** Photograph of the experimental setup. **c** Photograph of the metallic chamber with the sample placed inside. **d** Image of a sample wirebonded to a chip carrier and mounted on a chip holder with the flexible heater. **e** Photograph of the sample mounted on a chip holder with the flexible heater

Gas sensing characterizations were carried out under controlled atmosphere inside an electrically grounded chamber to limit external noise during measurements. The sample was wirebonded to a chip carrier with a flexible heater underneath (see Fig. 2e). The chip carrier was mounted on chip holder and placed inside an aluminum chamber with electrical feedthroughs and gas inlet and outlet (see Fig. 2c). Surface charge was recorded over time, upon constant flow of 1 L/min and at atmospheric pressure. The gas chamber has a volume of 160 mL and has been designed to minimize turbulences, and the entire volume is regenerated within 10 s at the operation total flow of 1 L/min. Mass flow controllers (MFCs) were used to adjust H_2_ concentration in synthetic air (80 % N_2_ and 20 % O_2_) used as a background gas. The whole measurement setup, shown in Fig. 2b, is interfaced with a LabVIEW program for MFCs control and data acquisition. A schematic representation of the experimental setup is shown in Fig. 2a. To allow sample recovery and stabilize, between each two measurements, the chamber is purged under synthetic air flow of 1 L/min for at least 1 h at the temperature of subsequent measurement point. During each measurement, the sample was first soaked in background gas flow for 1000 s, to acquire a baseline signal, before injecting H_2_ at a desired concentration during 1000 s to acquire the response signal after reaching the steady state. Then, H_2_ feeding was switched off, and the sample was under background gas flow for 1000 s. The surface charge variation is the difference between the two acquired values. Material sensitivity is defined as the surface charge variation per unit concentration variation and unit surface. Charge measurements were carried out in the operation temperature range from 80 to 190 °C at constant H_2_ concentration of 4 % and for different concentrations ranging from 0.5 to 4 % at an operating temperature of 130 °C.

Experimental data presented in Figs. 4 and 5 are mean values of Pt response over five subsequent repeated measurements. Mean values and standard deviations, represented by bar errors in Figs. 4 and 5, have been estimated by statistical analysis over the five repeated measurements.

## Results and Discussion

**Fig. 3:**
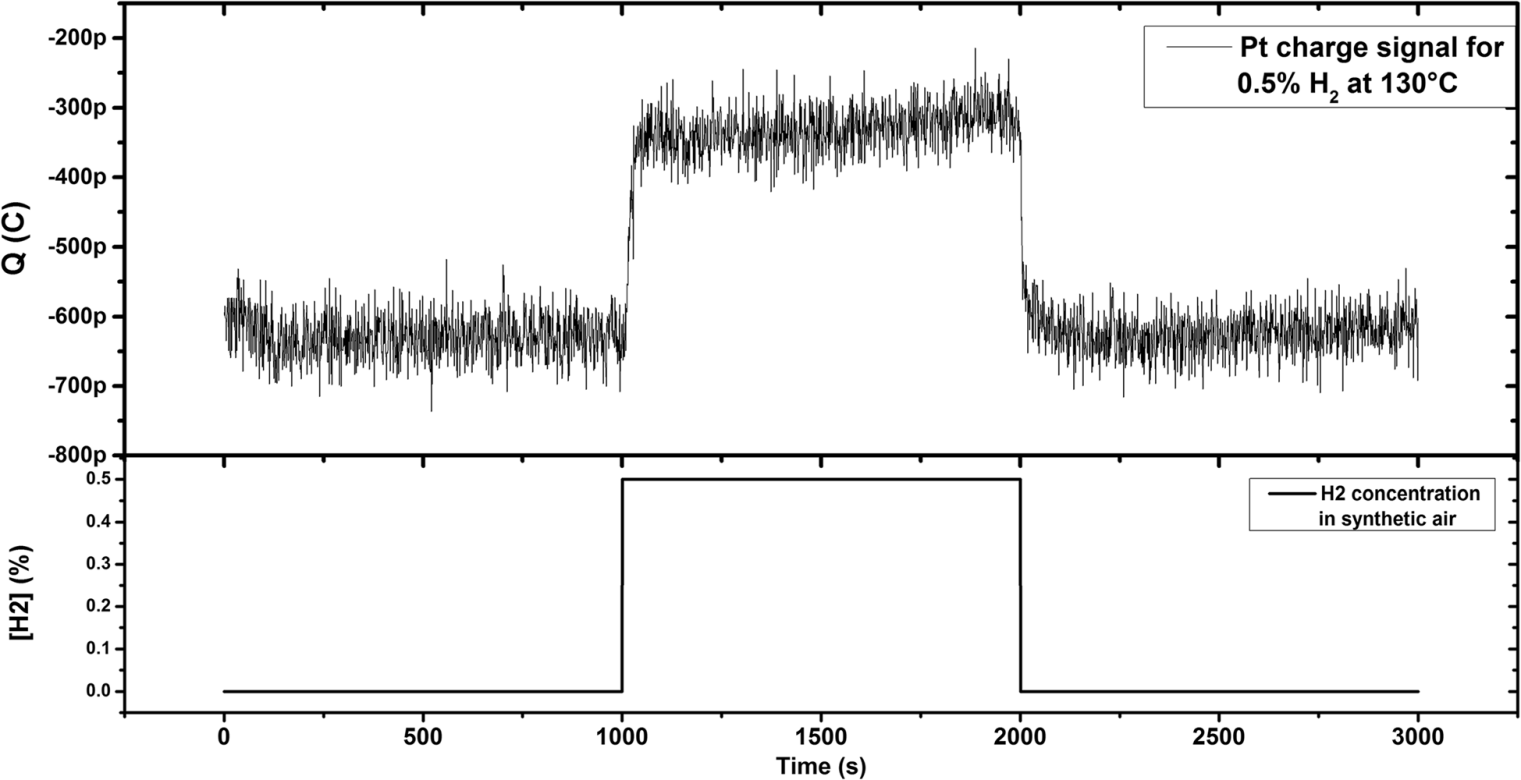
Time response of platinum layer to injection of 0.5 % hydrogen in synthetic air maintained for 1000 s at 130 °C

A typical response of platinum in terms of surface charge to 0.5 % hydrogen injection at 130 °C is shown in Fig. 3. At 130 °C, Pt exhibited clear and fast response in the presence of H_2_. But it has been difficult to distinguish a clear response over the signal noise for measurements carried out at 130 °C at H_2_ concentration lower than 0.5 %. This is due to the signal noise which was larger or comparable to the response signal. The standard deviation of the recorded signal noise was estimated by statistical signal processing to be equal to 30 pC at 130 °C. The noise source may come from the instrument electronics but also from the sample because it has been observed that the noise signal standard is temperature dependent. For this reason, we applied our technique to investigate Pt sensitivity at varying concentrations in synthetic air at 130 °C.

**Fig. 4:**
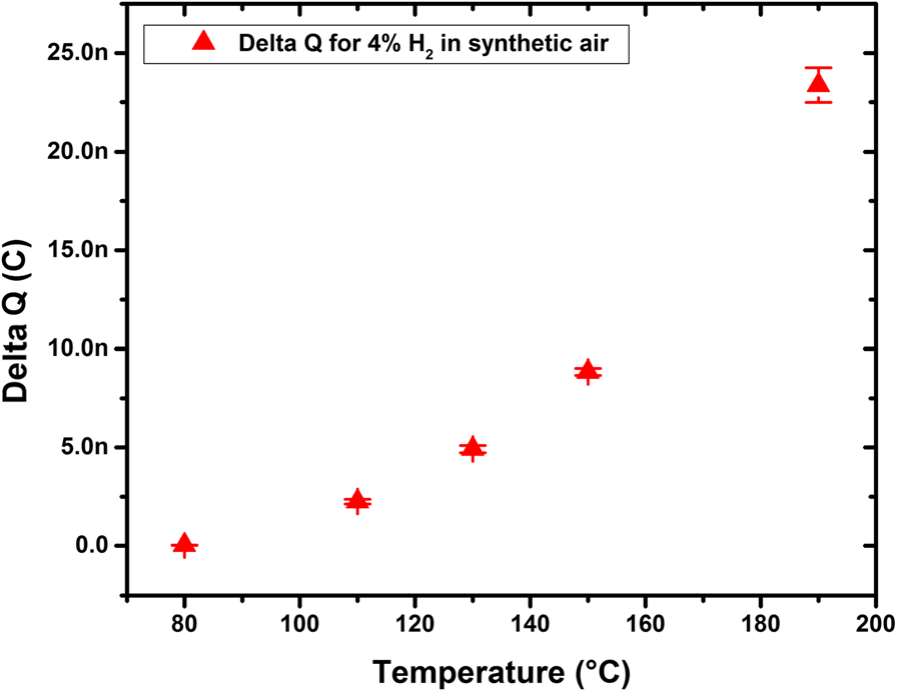
Surface charge variation at varying temperature of a 2.6 × 5 mm^2^ surface-platinum layer at hydrogen concentration of 4 %

**Fig. 5:**
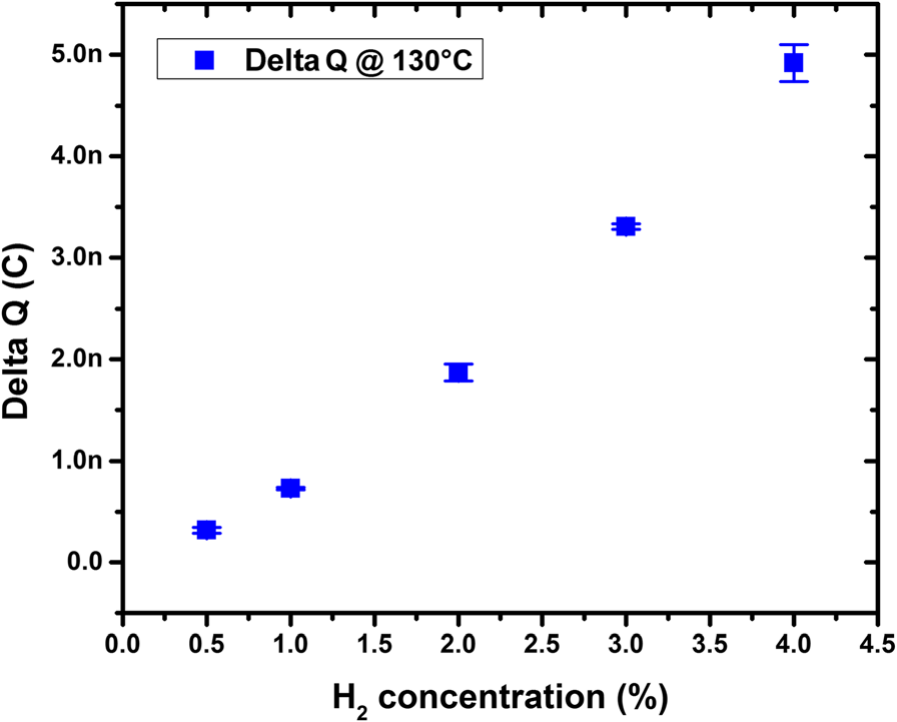
Platinum layer response in terms of surface charge variation as a function of hydrogen concentration at 130 °C

At temperature below 80 °C, the Pt exhibited either instability or no response probably due to moisture environment. For this reason, we have investigated the range of 80–190 °C for this work. The surface charge variation with respect to operation temperature for H_2_ concentration of 4 % is shown in Fig. 4 and appears to increase strongly above 120 °C. The range 130–190 °C is known to be the sensitivity temperature range of Lundstrom FETs [3, 6, 15, 16]. Induced surface charge variations at varying hydrogen concentration at 130 °C are presented in Fig. 5. We obtained a linear material response, in terms of surface charge variation, to varying H_2_ concentration from 0.5 to 4 %, which is in good agreement with the theory. The induced surface charge is proportional to the adsorbed hydrogen atom concentration, and according to literature [3, 4, 15], the density of adsorbed hydrogen is assumed to be proportional to hydrogen partial pressure in the absence of poisoning. From experimental data presented in Fig. 5, the sensitivity of the sensing layer per surface area is estimated to be equal to 96 pC per % per mm^2^.

The equation describing the hydrogen chemisorption at the surface of Pt can be written as:

1 $$ {H}_{2\mathrm{gas}}\underset{K_{\mathrm{des}}}{\overset{K_{\mathrm{ads}}}{\rightleftharpoons }}2\ {H}_a $$

In presence of oxygen, we assume that adsorbed hydrogen and oxygen atoms may react and form H_2_O. This interaction is given by the following equations:

2 $$ {O}_{2\mathrm{gas}} + 2\ {H}_a\overset{C_1}{\to }2\ O{H}_a $$

3 $$ O{H}_a + \kern0.5em {H}_a\overset{C_2}{\to }\ {H}_2{O}_{\mathrm{gas}} $$

where index gas refers to gas phase species and *a* to atomic adsorbed species; *K*_ads_ and *K*_des_ stand for, respectively, adsorption reaction and desorption reaction constants of hydrogen chemisorption on platinum (Eq. 1). *C*_1_ and *C*_2_ denote reaction constants of, respectively, Eqs. 2 and 3. Back reaction of Eq. 2 is neglected owing to the large enthalpy of forward reaction.

Possible other reactions that can take place at the Pt surface between nitrogen and adsorbed hydrogen adatom can be described by the following equations:

4 $$ {N}_{2\mathrm{gas}} + 2\ {H}_a\overset{K_1}{\to }2\ N{H}_a $$

5 $$ 2N{H}_a + \kern0.5em {H}_a\overset{K_2}{\to }\ N{H}_{2a} $$

6 $$ N{H}_{2a} + \kern0.5em {H}_a\overset{K_3}{\to }\ N{H}_{3\mathrm{gas}} $$

where all back reactions of Eqs. 4, 5, and 6 have been neglected; *K*_1_, *K*_2_, and *K*_3_ denote reaction constants of, respectively, Eqs. 4, 5, and 6.

Surface coverage rate *Θ* is defined as the ratio of adsorbed hydrogen concentration [H_a_] by the total density (concentration) of available surface sites for chemisorption, occupied or unoccupied, [S_t_]:

7 $$ \varTheta =\frac{\left[{H}_a\right]}{\left[{S}_t\right]} $$

Similarly *υ*, *φ*, and *ψ* denote surface fractional coverage of OH_a_, NH_a_, and NH_2a_, respectively, assuming the number of adsorption sites to be the same. Using the equation of H_2_ chemisorption and coverage rate definition, the rate equation for H_2_ chemisorption will be:

8 $$ \begin{array}{l}\frac{d\varTheta }{dt}={K}_{\mathrm{ads}}\ {P}_{H_2}\ {\left(1-\varTheta \right)}^2-{K}_{des}\ {\varTheta}^2-{C}_1\ {P}_{O_2}\ {\varTheta}^2\ {\left(1-\upsilon \right)}^2-{C}_2\ \varTheta\ \upsilon - \\ {}{K}_1\ {P}_{N_2}\ {\varTheta}^2{\left(1-\varphi \right)}^2-{K}_2\kern0.5em \varTheta\ {\varphi}^2\ \left(1-\psi \right)-{K}_3\ \varTheta\ \psi \end{array} $$

9 $$ \frac{d\upsilon }{dt}={C}_1\ {P}_{O_2}\ {\varTheta}^2\ {\left(1-\upsilon \right)}^2-{C}_2\ \varTheta\ \upsilon $$

10 $$ \frac{d\varphi }{dt}={K}_1\ {P}_{N_2}\ {\varTheta}^2\ {\left(1-\varphi \right)}^2-{K}_2\ \varTheta\ {\varphi}^2\ \left(1-\psi \right) $$

11 $$ \frac{d\psi }{dt}={K}_2\ \varTheta\ {\varphi}^2\ \left(1-\psi \right)-{K}_3\ \varTheta\ \psi $$

where ${P}_{H_2},\ {P}_{O_2},\ \mathrm{and}\ {P}_{N_2}$ are partial pressure of hydrogen, oxygen, and nitrogen. Factors like (1 − *Θ*)^2^ and *Θ* ^2^ arise from the fact that two adsorption sites are involved in adsorption-desorption reaction of Eq. 1. At equilibrium (steady state) $\frac{d\varTheta }{dt}=0,$ $\frac{d\upsilon }{dt}=0,$ $\frac{d\varphi }{dt}=0,$ and $\frac{d\psi }{dt}=0$ and assuming that *υ* ≪ 1 and *φ* ≪ 1, the steady-state coverage rate is given by:

12 $$ \frac{\varTheta }{1-\varTheta }={\left(\frac{K_{\mathrm{ads}}\ {P}_{H_2}}{2\ {C}_{1\ }{P}_{O_2} + 3\ {K}_{1\ }\ {P}_{N_2} + {K}_{\mathrm{des}}}\right)}^{1/2} $$

**Fig. 6:**
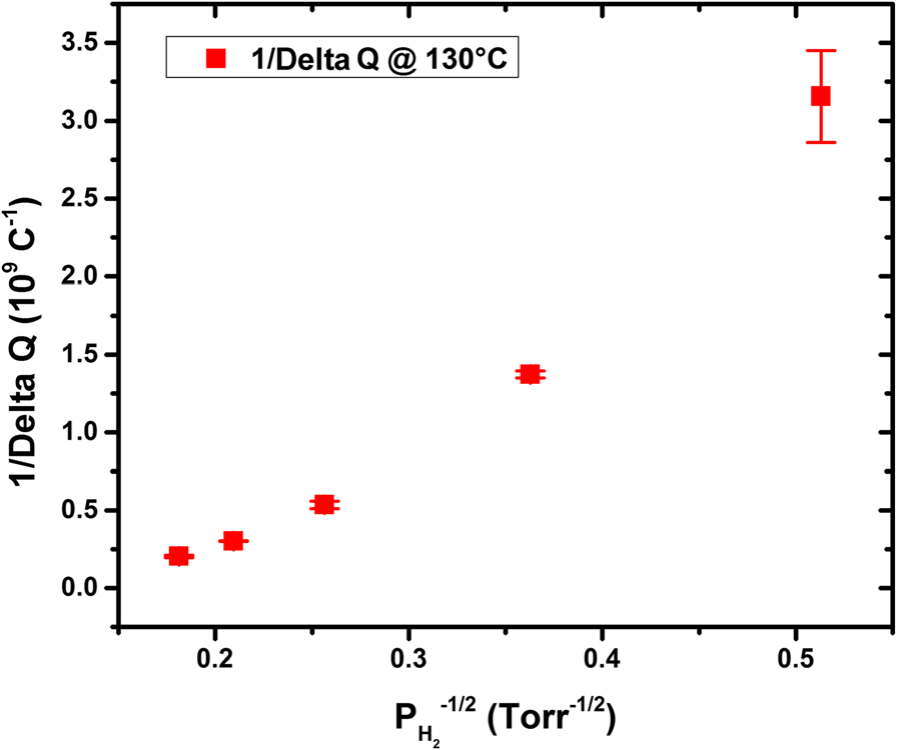
Plot of 1/Δ*Q* versus ${P_{H_2}}^{-1/2}$ at 130 °C

Surface charge variation ∆*Q* is assumed to be proportional to coverage rate *Θ*. Assuming that *Θ* = 1, where all adsorption sites are occupied, which corresponds to the maximum variation in the surface charge of Pt and using Eq. 12, we have:

13 $$ \frac{1}{\varDelta Q}=\frac{1}{\varDelta Q \max }\ {\left(\frac{2\ {C}_{1\ }{P}_{O_2} + 3\ {K}_{1\ }\ {P}_{N_2} + {K}_{\mathrm{des}}\kern0.5em }{K_{\mathrm{ads}}\ {P}_{H_2}}\right)}^{1/2} + \frac{1}{\varDelta Q \max }, $$

where ∆*Q*_max_ stands for maximum surface charge variation due to the of hydrogen on all adsorption sites. Equation 13 implies that *1*/∆*Q* is proportional to ${\left({P}_{H_2}\right)}^{-1/2}$. A plot of experimental results of *1*/∆*Q* versus ${\left({P}_{H_2}\right)}^{-1/2}$ in Fig. 6 shows a linear dependence, which is in accordance with the Eq. 13. From the slope and intercept of linear fit of data presented in Fig. 6, ∆*Q*_max_ could be estimated. Although the linear curve of *1*/∆*Q* versus ${\left({P}_{H_2}\right)}^{-1/2},$ the intercept is found negative. We strongly suspect a negative offset to measurements (instrument systematic error). For instance, the voltage burden of the electrometer coulombmeter function could possible contribute a negative offset to measurements, presented in Fig. 5, which in return affects the results depicted in Fig. 6. ∆*Q*_max_ correlated to material sensitivity calibration curve (Δ*Q* Versus H_2_ concentration) would give an approximation of H_2_ concentration threshold above which the material response saturates. This is with great interest for the estimation of sensitivity concentration range.

## Conclusions

Sensitivity investigation by surface charge measurement can be applied to any type of sensing material where chemisorption or physisorption induce a surface charge variation. This technique allows the estimation of a maximum surface charge variation which corresponds to the saturation of all possible adsorption sites. Also, sensitivity can be estimated and used for sensing layer design and sizing with regard to transducer transfer function and target concentration range.

## Abbreviations

FET: Field-effect transistor
MFC: Mass flow controller
MOSFET: Metal oxide semiconductor field-effect transistor
RT: Room temperature
SG-FET: Suspended gate-field effect transistor
